# Toxic epidermal necrolysis following treatment of pseudotumour cerebri: a case report

**DOI:** 10.1186/1757-1626-2-9402

**Published:** 2009-12-29

**Authors:** Mohamed El Ghonemi, Hesham R Omar, Rania Rashad, Jaya Kolla, Devanand Mangar, Enrico Camporesi

**Affiliations:** 1 Department of Critical Care, Cairo University, Cairo, Egypt; 2 Department of Cardiology, Cairo University, Cairo, Egypt; 3 Department of Family Medicine, University of Tennessee, Tennessee, USA; 4 Department of Anesthesiology, Tampa General Hospital , Tampa, Florida, USA; 5 Florida Gulf to Bay Anesthesiology Associates, PA, Tampa, Florida, USA; 6 Department of Surgery/Anesthesiology, University of South Florida, Tampa, Florida, USA; 7 Department of Molecular Pharmacology and Physiology, University of South Florida, Tampa, Florida, USA

## Abstract

Toxic Epidermal Necrolysis and Steven-Johnson syndrome are entities on a spectrum of cutaneous reactions that usually occur as an idiosyncratic reaction to certain drugs. The distinction between TEN and SJS is based on the percentage of skin involved with SJS being less than 10% and TEN being more than 30%. They exhibit severe skin blistering and sloughing with mucosal involvement and can be fatal in many cases. Discontinuation of the offending agent is mandatory together with reduction of skin manipulation and avoiding infection. Plasmapharesis, intravenous immunoglobulins and immunosuppressants have been used with conflicting results. In this manuscript we are describing a 22 year old female patient from Egypt who presented with severe skin sloughing with mucosal involvement following carbamazepine therapy. The incriminated drug was discontinued and urgent life saving therapy in the form of broad spectrum antibiotic, immunosuppression with cyclophosphamide, Intensive Care Unit admission and nursing care was started followed by dramatic response. The clinical presentation, pathogenesis and modalities of treatment will be described in details.

## Introduction

TEN and SJS are severe, acute and rare mucocutaneous diseases that are usually elicited by drugs. Many different groups of drugs can cause TEN, including anticonvulsants, nonsteroidal anti-inflammatory drugs, allopurinol and antibiotics. TEN is characterized by extensive blistering, full-thickness necrosis, and destruction of the epidermis. TEN and SJS are the same disease spectrum that can present with differences in severity and area of involvement. SJS is less extensive and affects less than 10% of the body surface area while TEN involves more than 30% BSA. The mortality rate of SJS is up to 5%, while the mortality among patients with TEN may exceed 30%. TEN patients should be treated in a burn center or intensive care unit. No optimal treatment for SJS and TEN has been developed. But recently, IVIG has been suggested for patients with TEN. This case report aims to sensitize readers to the possibility of the occurrence of this rare complication following carbamazepine therapy and the successful use of cyclophosphamide to dramatically cure the condition.

## Case presentation

A 22-year-old Caucasian female with a BMI of 35 kg/m2 from Egypt, with no past medical history of clinical significance presented to the outpatient clinic one month after a normal delivery with severe headache and blurring of vision. Fundus examination showed evidence of bilateral papilledema, brain CT scan was normal and the patient was diagnosed with benign intracranial hypertension. She underwent therapeutic CSF aspiration and was maintained on carbamazepine and acetazolamide to decrease intracranial pressure.

**Figure 1:**
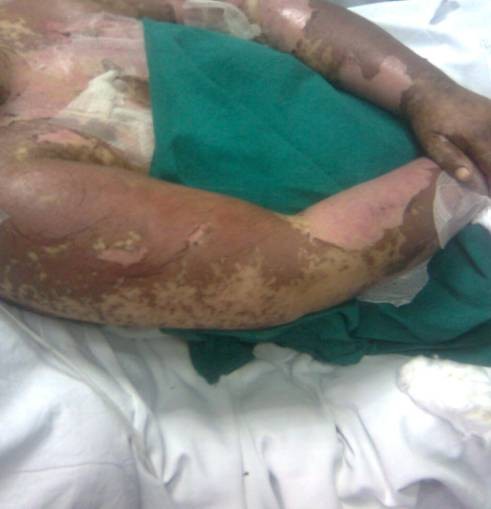
**Toxic epidermal necrolysis with generalized sloughing of the epidermis involving more than 30% of the body surface area**.

After 5 days of carbamazepine therapy the patient started to complain of generalized skin eruptions in the form of irregularly shaped macules distributed on the face, trunk, upper and lower limbs as illustrated in figure 1. This was followed by grayish discoloration and mottling of the skin and mucous membranes. Mucosal involvement was noticed in the form of conjunctival injection and oral lesions.

The patient was admitted to the Intensive Care Unit with high fever, extensive skin sloughing, clinical evidence of dehydration and severe pain mandating continuous morphine infusion. Skin lesions showed a positive Nikolsky sign and ophthalmological examination revealed bilateral conjunctivitis. Initial workup revealed clinical and laboratory evidence of sepsis in the form of hypotension, leukocytosis, elevated Erythrocyte sedimentation rate, metabolic acidosis, high serum lactate level and otherwise normal biochemical profile. Skin lesions were pathognomonic of Toxic Epidermal Necrolysis (TEN) with more than 30% skin involvement. Detailed history taking revealed the recent introduction of carbamazepine therapy for treatment of pseudotumour cerebri. Drug induced TEN was suspected and carbamazepine was withdrawn.

The patient was managed with Lactated ringer solution together with the use of sterile skin dressings to reduce pain and risk of infection. The patient was started on immunosuppressant therapy in the form of cyclophosphamide. Blood and skin cultures were positive for pseudomonas and patient was started on imipinem/cilastatin.

**Figure 2:**
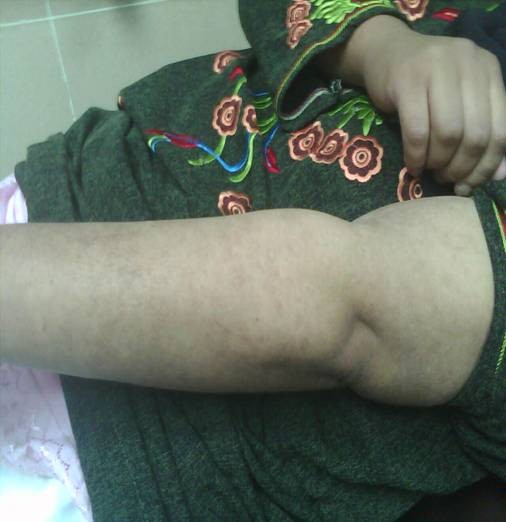
**Demonstrating complete resolution of the skin lesions following cyclophosphamide therapy**.

Dramatic improvement in the patient condition was noticed after one week of cyclophosphamide therapy with complete resolution of the skin lesions, mucosal involvement and pain as shown in figure 2. Metabolic acidosis, leukocytosis and fever resolved together with the normalization of serum lactate level. Ophthalmological follow up revealed resolution of the conjunctivitis with no evidence of scarring.

## Discussion

Alan Lyell described TEN in 1956, describing the condition as "an eruption resembling scalding of the skin [1]. TEN is characterized by epidermal loss suggestive of severe scalding. In that same year, Lang and Walker also observed a patient with TEN [2], which was originally described by Debre et al in 1939 [3].

TEN is a rare disease. The incidence in adults is estimated to be between 0.4 and 1.2 cases per 1 million people per year [4-9]. Carbamazepine caused SJS/TEN in a frequency of 14 per 100000 users [10]. Death often occurs early in the course of the disease with sepsis being the most frequent cause. *Pseudomonas aeruginosa *and *Staphylococcus aureus *are the predominant organisms involved. The mortality rate of SJS is up to 5%, while the mortality among patients with TEN may exceed 30%. Mortality is increased significantly in patients who are at the extremes of age and in relation to the percentage of denuded skin [8,11].

The actual pathophysiologic mechanism of TEN remains uncertain; however, it seems apparent that an exposure to certain drugs plays a significant role in triggering the disease process. These drugs include anticonvulsants, such as phenobarbital, phenytoin, valproic acid; antibiotics such as sulfonamides, aminopenicillins, quinolones, and cephalosporins; anti-inflammatory drugs; acetaminophen; allopurinol; and corticosteroids [12], The relatively new antiepileptic drug; lamotrigine has also been described as a triggering factor. The incidence of cross reactivity among the aromatic anticonvulsants: carbamazepine, phenytoin, phenobarbital, which share an arene oxide intermediary, is greater than 75% [13].

Carbamazepine is being increasingly prescribed for various indications specially pain control and this may be a reason for the increased incidence of SJS/TEN due to carbamazepine. This case report describes the occurrence of TEN after carbamazepine therapy used to treat a patient with psudotumour cerebri. In 2004, a genetic marker, the human leukocyte antigen HLA-B 1502, was reported to be strongly associated with carbamazepine-induced SJS or TEN in Han Chinese [14].

TEN is believed to be an immune-related cytotoxic reaction aimed at destroying keratinocytes that express drug related antigens. It mimics a hypersensitivity reaction, with its characteristic delayed reaction to an initial exposure and an increasingly rapid reaction with repeated exposure. Explanations for the generalized nature of TEN include the belief that tumor necrosis factor-α (TNF-α) which is derived from macrophages and keratinocytes is over expressed in the epidermis and play an important role in epidermal destruction directly through apoptosis, indirectly through stimulating cytotoxic T lymphocytes, or both.

Treatment of TEN focuses on early withdrawal of the causative agent and referral of the patient to a burn center for critical care and appropriate wound management. Local care of the denuded skin areas is of utmost importance to avoid infection. Bacterial cultures should be withdrawn and antibiotics started with the first sign of infection to prevent septicemia

Ophthalmological follow-up is recommended to prevent conjunctival scarring. Systemic therapy for TEN remains controversial with no standard guidelines available. The use of systemic corticosteroids is very controversial and a consensus has not been reached so far; plasmapharesis, immunoglobulins and immunosuppressant drugs have been tried with non-conclusive results [15-17]. In our patient the early use of cyclophosphamide resulted in dramatic improvement of skin sloughing and mucosal damage.

## Conclusion

TEN is a rare but serious disorder that most commonly occurs secondary to specific drug exposure. TEN is a generalized disease that involves skin and mucous membranes of a large body surface area. Skin infection and sepsis are the most common complications and care must be given for prevention and early treatment of sepsis. Many treatment modalities have been used including plasmapharesis, immunoglobulins and immunosuppressants but with conflicting results. In our patient the early use of cyclophosphamide resulted in a rapid dramatic improvement of the skin lesions together with the resolution of the signs of sepsis. This case report aims to sensitize readers to the possibility of the occurrence of this rare complication following carbamazepine therapy. Adequate awareness about the culprit drugs in this life threatening complication will help physicians in preventing them by judicious use of these drugs.

## Abbreviations

CT: computed tomography; IVIG: intravenous immunoglobulin; SJS: Steven Johnson syndrome; TEN: toxic epidermal necrolysis.

## Consent

Written informed consent was obtained from the patient for publication of this case report. A copy of the written consent is available for review by the Editor-in-Chief of this journal.

## Competing interests

The authors declare that they have no competing interests.

## Authors' contributions

ME, HO and RO were responsible for drafting the manuscript. RO and ME were responsible for diagnosis of the case. EC, JK and DM have made critical revisions to the manuscript. All authors have read and approved the final manuscript.
